# Cholesterol remnants and triglycerides are associated with decreased myocardial function in patients with type 2 diabetes

**DOI:** 10.1186/s12933-016-0454-x

**Published:** 2016-09-22

**Authors:** Peter Godsk Jørgensen, Magnus Thorsten Jensen, Tor Biering-Sørensen, Rasmus Mogelvang, Søren Galatius, Thomas Fritz-Hansen, Peter Rossing, Tina Vilsbøll, Jan Skov Jensen

**Affiliations:** 1 Department of Cardiology, Herlev and Gentofte Hospital, University of Copenhagen, Kildegårdsvej 28, 2900 Hellerup, Denmark; 2 Faculty of Health Sciences, Institute of Clinical Medicine, University of Copenhagen, Blegdamsvej 3B, 2200 Copenhagen N, Denmark; 3 Steno Diabetes Center, Niels Steensens Vej 2-2, 2820 Gentofte, Denmark; 4 Faculty of Health, Aarhus University, Nordre Ringgade 1, 8000 Aarhus C, Denmark; 5 Center for Diabetes Research, Herlev and Gentofte Hospital, University of Copenhagen, Kildegårdsvej 28, 2900 Hellerup, Denmark

**Keywords:** Type 2 diabetes, Lipids, Echocardiography

## Abstract

**Background:**

Recently, genetic studies have suggested a causal relationship between cholesterol remnants and ischemic heart disease. We aimed to determine whether cholesterol remnants and its marker, triglyceride levels, are associated with cardiac function as determined by sensitive echocardiographic measures in a population of patients with type 2 diabetes.

**Methods:**

Comprehensive echocardiography including 2D-speckle tracking echocardiography was performed on a representative sample of 924 patients with type 2 diabetes—730 of whom were treated with statins. These were recruited from two large secondary care centers.

**Results:**

In multivariable analyses, triglycerides and cholesterol remnants were not associated with left ventricular ejection fraction, but with subtle measures of systolic function, including global longitudinal strain by speckle tracking and longitudinal displacement by tissue Doppler echocardiography: global longitudinal strain [0.33 % (0.14), p = 0.02 per doubling in cholesterol remnants and 0.28 % (0.13), p = 0.03 per doubling in triglyceride levels] and with longitudinal displacement [−0.25 mm (0.10), p = 0.01 per doubling in cholesterol remnants and −0.25 mm (0.09), p = 0.005 per doubling in triglyceride levels]. Subgroup analyses of patients receiving statin therapy and patients without known heart disease revealed similar results, but the association was not present in patients with known heart disease.

**Conclusion:**

In patients with type 2 diabetes, subtle decrease in left ventricular function is present with increasing levels of cholesterol remnants and triglyceride levels indicating an effect of these on cardiac function that is not detectable by conventional echocardiography.

**Electronic supplementary material:**

The online version of this article (doi:10.1186/s12933-016-0454-x) contains supplementary material, which is available to authorized users.

## Background

In patients with type 2 diabetes, advances in medical treatment—including aggressive lipid-lowering therapy with statins and life-style interventions—have led to a decrease in mortality from cardiovascular disease [1–3]. However, patients with type 2 diabetes are still prone to cardiovascular disease and with an increasing number of patients with type 2 diabetes, the burden of cardiovascular disease persists [4]. The residual risk may in part be attributable to lipoproteins other than LDL-cholesterols that are the target of statin therapy, and recently, genetic studies have suggested a causal relationship between cholesterol remnants and ischemic heart disease [5, 6]. Cholesterol remnants—defined as cholesterol content of the largest, triglyceride-rich proteins, VLDL, IDL and chylomicrons—are reversely related to HDL cholesterol and have gained renewed interest in the light of the disappointing results of therapies aimed at HDL cholesterol.

Remnant cholesterol possibly exerts its effect on the diabetic myocardium by at least 2 mechanisms. First, the remnant lipoproteins can enter the intima of the coronary arteries causing accumulation of cholesterol [7, 8] ultimately leading to at least partly obstructive disease affecting mainly the subendocardial myocardial fibers that are most susceptible to decreased coronary flow and are the main myocardial fibers responsible for left ventricular longitudinal function [9]. Second, action of the enzyme lipoprotein lipase on the surface of the remnant particle leads to liberation of free fatty acids in the proximity of the myocardial cells [10] which can lead to accumulation of free fatty acids and triglycerides in the myocytes. Here, the substrate of metabolism is altered to almost complete reliance of fatty acids, which in turn by complex pathways is thought to lead to diabetic cardiomyopathy [11, 12]. In accordance, triglycerides—which are markers of cholesterol remnants [6, 13]—have been shown to predict heart failure in patients with diabetes, but not in patients without diabetes [14, 15].

While cholesterol remnants are considered a risk factor for ischemic heart disease, their relation to myocardial structure and function is unknown. Therefore, we examined the effect of cholesterol remnants and triglycerides on myocardial structure and function in a population of patients with type 2 diabetes using comprehensive echocardiography including 2D speckle tracking echocardiography. This is a sensitive method used to identify subtle changes in cardiac function that we have shown to be reduced in patients with type 2 diabetes [16] and has been shown to predict cardiovascular events in these patients [17]. In addition, to address if statin treatment modifies the association of cholesterol remnants and triglyceride with myocardial structure and function, we performed a subgroup analysis on patients already receiving statin therapy.

## Methods

The Thousand & 2 echocardiographic study was initiated in 2011, where a representative sample of patients from two large secondary care centers (The Steno Diabetes Center and Center for Diabetes Research, Herlev and Gentofte Hospital, University of Copenhagen) in the Copenhagen region were invited to participate. Details are described elsewhere [18]. In brief, from November 2011 through to December 2013, 2158 patients were invited and 1030 participated (participation rate 47.8 %). Prior to the study visit, a questionnaire was sent to the patients with among others questions on current medication, prior heart disease (myocardial infarction, percutaneous coronary intervention, coronary artery bypass grafting, congestive heart failure and atrial fibrillation), prior stroke and peripheral artery disease, family history of coronary heart disease, smoking habits, height and weight. At the visit, the questionnaire was reviewed with the patient by PGJ. In the present study, we excluded patients with atrial fibrillation during the echocardiographic examination and/or severe valve disease/previous heart valve surgery (n = 96) as well as patients where neither cholesterol remnants nor triglyceride levels (n = 10) were available. Blood pressure was measured in the supine position after at least 15 min of rest. Body mass index (BMI) was defined as weight (kg)/height (m)^2^.

### Cholesterol remnants and triglyceride

LDL-cholesterol, HDL-cholesterol and total-cholesterol were measured in routine blood samples using standard hospitals assays at each visit at Center for Diabetes research, Herlev and Gentofte Hospital, University of Copenhagen or at the visit at Steno Diabetes Center preceding the study visit. In Denmark, non-fasting measurements of lipids is the standard, however, in a subset of the patients (14 %), only fasting lipids were available. Direct measurement of cholesterol remnants is difficult, but a simple approximation was obtained by calculating the sum of VLDL, IDL and chylomicrons with the formula cholesterol remnants = total-cholesterol − HDL-cholesterol − LDL-cholesterol. This method is easy with the use of standard lipid measurements and has been used in previous studies [5, 6, 13, 19].

### Other laboratory analyses

Hemoglobin A_1c_ and creatinine levels were obtained from routine blood samples at either center as decribed above. Urine albumin/creatinine ratio or 24 h urine albumin excretion rate is performed at least annually at both centers. Microalbuminuria was defined as urine albumin/creatinine ratio between 30 and 300 mg/g or urine albumin excretion rate between 30 and 300 mg/day, and macroalbuminuria as urine albumin/creatinine ratio above 300 mg/g or urine albumin excretion rate above 300 mg/day in two consecutive measurements.

### Echocardiography

Echocardiography was performed with General Electrics, Vivid 7 and Vivid 9 (Vingmed ultrasound, Horten, Norway) and digitally stored. Three consecutive heart cycles were recorded for each view. The Thousand&2 echocardiograms (>95 %) were made by PGJ and all offline analyses were performed using General Electrics EchoPac software BT13 by on physician (PGJ). Chamber quantification was done in accordance with the recommendations of the European Association of Echocardiography and the American Society of Echocardiography [20]. Left ventricular (LV) mass was calculated using the formula LV mass = 0.8 × {1.04[(LV internal diameter + posterior wall thickness + septal wall thickness)^3^ − (LV internal diameter)^3^]} + 0.6 g and indexed according to height^2.7^. Left atrial (LA) end-systolic and end-diastolic volumes were calculated using the area-length method, where left atrial volume = 8/3 × π (LA area in 4 chamber view × LA area in 2 chamber view/shortest long axis length in either view) and indexed to body surface area. Mitral inflow velocities [peak early (E) and peak atrial (A)] and deceleration time of the E wave were measured in the 4-chamber view using pulsed-wave Doppler with the sample volume placed between the tips of the mitral valve leaflets. Early diastolic myocardial velocity (e’) was measured in 4-chamber view with pulsed-wave tissue velocity Doppler with the sample volume placed in the septal and lateral mitral annulus.

### Left ventricular systolic function

LV ejection fraction was assessed using Simpson’s biplane method. Speckle tracking echocardiography was performed on 2D grey scale recordings of 4-, 2- and 3-chamber apical views and analyzed off-line. In brief, the software identified speckles within a specified part of the myocardium and tracked these from frame to frame. A semi-automated function traced the myocardium in the beginning of the systole and allowed for manual modification of the region of interest to ensure only speckles in the myocardium were tracked throughout the cardiac cycle. Visual inspection of the tracking curves ensured accurate tracking of the speckles and segments where the tracking was assessed inadequate were excluded from the analyses. Doing this for each segment of the left ventricle a measurement of longitudinal function or deformation expressed as peak systolic strain was obtained for each segment and for 3 layers of each segment. In the present study, midmyocardial strain was used and global longitudinal strain (GLS) was calculated as the average peak systolic strain of the 18 midmyocardial segments. If accurate tracking was obtained in less than 12 of the 18 midmyocardial segments because of poor image quality, the echocardiogram was excluded from the analyses (15.3 % of the examinations). As a sensitivity measure to complement the strain measurements of longitudinal cardiac function, longitudinal displacement obtained by color tissue Doppler imaging was performed. Longitudinal displacement was calculated as the average of the integral of the velocity curves during ejection phase of sample volumes placed in each of the mitral annular positions. This measure has previously been validated and demonstrated to be a very accurate measure of mitral annular plane excursion [21].

### Statistics

Both the distribution of the triglyceride levels and the cholesterol remnant levels were markedly skewed, so they were logarithmically transformed in the analyses using the binary logarithm [log_2_ (triglyceride) and log_2_ (cholesterol remnants)] after which both were normally distributed. Linear regression analyses were used to examine the effect of the logarithmically transformed levels of triglyceride and cholesterol remnants on measures of cardiac structure, systolic and diastolic function. Multivariable models were constructed with age, sex, hemoglobin A_1c_, body mass index, systolic blood pressure and albuminuria as covariates. P-values less than 0.05 on two-sided tests were considered significant. Statistics were calculated using R for Mac, version 2.15.3 (R Project for Statistical Computing, Vienna University of Economics and Business Administration, Wien, Austria).

## Results

A total of 924 patients were identified of whom 730 were receiving statin therapy. The study population’s clinical and echocardiographic characteristics are shown in Table 1 and Additional file 1: Table S1. Notably, both groups and in particular the patients receiving statin therapy had low LDL-cholesterol levels.

**Table 1:** Study population characteristics

	All	Receiving statin therapy
	n = 924	n = 730
*Clinical*		
Age (years)	65 [58, 70]	65 [59, 70]
Male sex, n (%)	597 (65)	486 (67)
Diabetes duration (years)	11 [6, 17]	11 [6, 17]
Body mass index (kg/m^2^)	29 [26, 33]	30 [27, 33]
Systolic blood pressure (mmHg)	136 (17)	135 (16)
Diastolic blood pressure (mmHg)	80 (11)	80 (10)
Known coronary heart disease n (%)	162 (18)	140 (19)
*Lipids*		
Total cholesterol (mmol/l)	4.1 [3.5, 4.8]	3.9 [3.4, 4.5]
Low density cholesterol (mmol/l)	2.0 [1.6, 2.6]	1.8 [1.5, 2.3]
High density cholesterol (mmol/l)	1.2 [1.0, 1.4]	1.2 [1.0, 1.4]
Cholesterol remnants (mmol/l)	0.8 [0.5, 1.1]	0.7 [0.5, 1.0]
Triglyceride (mmol/l)	1.7 [1.2, 2.4]	1.7 [1.2, 2.4]
*Other laboratory values*		
Albuminuria (%)	212 (23.3)	165 (22)
Microalbuminuria (%)	149 (16)	116 (16)
Macroalbuminuria (%)	63 (7)	49 (7)
Creatinine (μmol/l)	78 [65, 96]	78 [66, 96]
Haemoglobin A_1c_ (%)	55 [48, 66]	54 [48, 65]
Haemoglobin A_1c_ (mmol/mol)	7.2 [6.5, 8.2]	7.1 [6.5, 8.1]
*Medication*		
Statins, n (%)	730 (79)	–
Metformin, n (%)	670 (73)	552 (76)
DPP4 inhibitors, n (%)	88 (10)	71 (10)
Sulfonylurea, n (%)	142 (15)	107 (15)
Glucagon-like peptide-1 receptor agonists, n (%)	226 (25)	189 (26)
Insulin, n (%)	428 (46)	334 (46)
Beta blockers, n (%)	221 (24)	186 (26)
Angiotensin-converting enzyme inhibitors, n (%)	354 (38)	297 (41)
Angiotensin II receptor blockers, n (%)	359 (40)	298 (41)
Calcium antagonists, n (%)	294 (32)	250 (34)
Diuretics, n (%)	452 (49)	376 (52)
Antiplatelets, n (%)	607 (66)	508 (70)

Continuous traits are reported as mean (standard deviation) or median [interquartile range] in case of non-normal distribution

### Structural and diastolic measurements

Structural and diastolic echocardiographic findings of the entire population including patients not receiving statin therapy is shown in Tables 2 and 3. For all patients as well as the subgroup of patients receiving statin therapy, increasing levels of log_2_ (triglyceride) were associated with increasing LV mass, LV wall thicknesses and decreasing LA size in univariable analyses, but this association was attenuated in multivariable analyses except for LA size. Regarding dynamic diastolic measurements, measures of early diastolic filling, including peak E velocity, lateral and septal e’ were only significantly associated in multivariable adjusted analyses with both log_2_ (cholesterol remnant) and log_2_ (triglyceride). Regarding LDL-cholesterol, LV wall thicknesses were inversely related to increasing levels of log_2_ (LDL-cholesterol), but none of the diastolic measurements were associated with increasing levels of log_2_ (LDL-cholesterol), Additional file 1: Table S2. A subgroup analysis of patients with and without known coronary heart disease showed that the structural and diastolic changes associated with log_2_ (cholesterol remnant) and log_2_ (triglyceride) were driven by patients without known coronary heart disease. An exception was septal e’ that was significantly associated with log_2_(cholesterol remnants) in patients with known coronary heart disease whereas lateral e’ was associated with both log_2_ (cholesterol remnant) and log_2_ (triglyceride) in patients without coronary heart disease.

**Table 2:** Structural changes in relation to log_2_(cholesterol remnants) and log_2_(triglyceride)

	log_2_ (cholesterol remnants)				log_2_ (triglyceride)			
	Univariable		Multivariable		Univariable		Multivariable	
	β-coefficient(std error)	P value	β-coefficient (std error)	P value	β-coefficient (std error)	P value	β-coefficient (std error)	P value
*All patients*								
Left ventricular mass index (g/m^2.7^)	0.6 (0.5)	0.27	−0.5 (0.5)	0.36	1.1 (0.5)	*0.03*	−0.1 (0.5)	0.81
Interventricular septum diameter (mm)	0.21 (0.08)	*0.01*	0.01 (0.08)	0.90	0.26 (0.08)	<*0.001*	0.04 (0.07)	0.58
Left ventricular internal diameter (mm)	−0.21 (0.29)	0.47	−0.47 (0.30)	0.11	−0.04 (0.26)	0.89	−0.37 (0.28)	0.18
Posterior wall diameter (mm)	0.16 (0.07)	*0.03*	0.01 (0.07)	0.90	0.25 (0.07)	<*0.001*	0.07 (0.06)	0.28
Left atrial end systolic volume index (ml/m^2^)	−1.61 (0.39)	<*0.001*	−1.41 (0.41)	*0.001*	−1.29 (0.35)	<*0.001*	−1.19 (0.37)	*0.002*
*In patients receiving statin therapy*								
Left ventricular mass index (g/m^2.7^)	0.7 (0.6)	0.24	−0.3 (0.6)	0.56	1.1 (0.5)	*0.04*	−0.1 (0.5)	0.86
Interventricular septum diameter (mm)	0.22 (0.09)	*0.02*	0.04 (0.09)	0.67	0.29 (0.09)	<*0.001*	0.08 (0.08)	0.35
End diastolic internal diameter (mm)	−0.30 (0.33)	0.36	−0.58 (0.34)	0.08	−0.17 (0.30)	0.57	−0.53 (0.31)	0.09
Posterior wall diameter (mm)	0.17 (0.08)	*0.04*	0.04 (0.08)	0.62	0.26 (0.07)	<*0.001*	0.10 (0.07)	0.17
Left atrial end systolic volume index (ml/m^2^)	−1.73 (0.45)	<*0.001*	−1.47 (0.47)	*0.002*	−1.44 (0.40)	<*0.001*	−1.33 (0.43)	*0.002*

Multivariable model adjusted for age, sex, hemoglobin A_1c_, body mass index, systolic blood pressure and albuminuria

Italic text indicates P < 0.05

**Table 3:** Diastolic changes in relation to log_2_(cholesterol remnants) and log_2_(triglyceride) in patients with type 2 diabetes

	log_2_ (cholesterol remnants)				log_2_ (triglyceride)			
	Univariable		Multivariable		Univariable		Multivariable	
	β-coefficient (std error)	P value	β-coefficient (std error)	P value	β coefficient (std error)	P value	β-coefficient (std error)	P value
*All patients*								
Peak E velocity (m/s)	−0.01 (0.01)	0.12	−0.03 (0.01)	*0.006*	−0.01 (0.01)	0.09	−0.03 (0.01)	*0.002*
Peak A velocity (m/s)	−0.003 (0.01)	0.76	−0.001 (0.01)	0.93	−0.01 (0.01)	0.51	−0.003 (0.01)	0.72
E/A ratio	−0.01 (0.01)	0.48	−0.03 (0.01)	*0.05*	−0.01 (0.01)	0.53	−0.03 (0.01)	*0.03*
E deceleration time (ms)	2.8 (3.5)	0.41	3.3 (3.6)	0.36	0.8 (3.1)	0.81	1.5 (3.3)	0.65
Lateral e’ (cm/s)	−0.18 (0.12)	0.13	−0.25 (0.11)	*0.02*	−0.19 (0.11)	0.07	−0.27 (0.10)	*0.008*
Septal e’ (cm/s)	−0.12 (0.09)	0.19	−0.18 (0.08)	*0.03*	−0.10 (0.08)	0.22	−0.17 (0.08)	*0.03*
E/e’_mean_	0.11 (0.20)	0.58	0.01 (0.20)	0.97	0.08 (0.18)	0.65	−0.02 (0.18)	0.93
*Patients receiving statin therapy*								
Peak E velocity (cm/s)	−0.02 (0.01)	0.08	−0.03 (0.01)	*0.006*	−0.02 (0.01)	0.08	−0.03 (0.01)	*0.004*
Peak A velocity (cm/s)	−0.002 (0.01)	0.82	−0.0003 (0.01)	0.98	−0.01 (0.01)	0.57	−0.002 (0.01)	0.86
E/A ratio	−0.02 (0.01)	0.25	−0.03 (0.01)	*0.02*	−0.01 (0.01)	0.36	−0.03 (0.01)	*0.02*
E deceleration time (ms)	3.8 (3.9)	0.33	3.57 (4.11)	0.39	1.3 (3.5)	0.72	1.1 (3.7)	0.77
Lateral e’ (cm/s)	−0.07 (0.13)	0.59	−0.19 (0.13)	0.13	−0.10 (0.12)	0.37	−0.21 (0.11)	0.07
Septal e’ (cm/s)	−0.07 (0.10)	0.49	−0.17 (0.10)	0.08	−0.10 (0.09)	0.27	−0.18 (0.09)	*0.04*
E/e’_mean_	−0.10 (0.22)	0.65	−0.17 (0.22)	0.44	−0.06 (0.20)	0.78	−0.13 (0.20)	0.53

Multivariable model adjusted for age, sex, hemoglobin A_1c_, body mass index, systolic blood pressure and albuminuria

Italic text indicates P < 0.05

### Systolic measurements

Results from echocardiographic systolic measurements are shown in Table 4. For all patients as well as the subgroup of patients receiving statin therapy conventional measurement of systolic function in the form of ejection fraction by Simpson’s biplane method was not associated with neither log_2_ (cholesterol remnant) nor log_2_ (triglyceride). However, for all patients, GLS was both uni- and multivariably associated with both log_2_ (cholesterol remnants) and log_2_ (triglyceride). The same was the case for longitudinal displacement measured by color tissue Doppler, though the association with log_2_ (cholesterol remnants) attenuated in the subgroup analysis of patients receiving statin therapy. Thus, two independent measurements of both LV longitudinal function both indicated decreased function with increasing levels of log_2_ (cholesterol remnant) and log_2_ (triglyceride). Similar results were found in the subgroup of patients receiving statin therapy, though some of the results were only borderline significant. Regarding LDL-cholesterol, there was no association between log_2_ (LDL-cholesterol) and longitudinal function expressed as either GLS or longitudinal displacement, Additional file 1: Table S2.

**Table 4:** Systolic changes in relation to log_2_(cholesterol remnants) and log_2_(triglyceride) in patients with type 2 diabetes

	log_2_ (cholesterol remnants)				log_2_ (triglyceride)			
	Univariable		Multivariable		Univariable		Multivariable	
	β-coefficient (std error)	P value	β-coefficient (std error)	P value	β-coefficient (std error)	P value	β-coefficient (std error)	P value
*All patients*								
Ejection fraction (%)	−0.7 (0.4)	0.09	−0.4 (0.4)	0.31	−0.6 (0.4)	0.09	−0.3 (0.4)	0.42
Global longitudinal systolic strain (%)	0.40 (0.13)	*0.003*	0.33 (0.14)	*0.02*	0.34 (0.12)	*0.004*	0.28 (0.13)	*0.03*
Longitudinal displacement (mm)	−0.25 (0.09)	*0.007*	−0.25 (0.10)	*0.01*	−0.25 (0.08)	*0.003*	−0.25 (0.09)	*0.005*
*Patients receiving statin therapy*								
Ejection fraction (%)	−0.7 (0.4)	0.12	−0.4 (0.5)	0.43	−0.5 (0.4)	0.18	−0.1 (0.4)	0.72
Global longitudinal systolic strain (%)	0.38 (0.15)	*0.01*	0.32 (0.16)	*0.05*	0.33 (0.14)	*0.01*	0.29 (0.15)	0.05
Longitudinal displacement (mm)	−0.20 (0.11)	0.07	−0.20 (0.11)	0.06	−0.22 (0.09)	*0.02*	−0.22 (0.10)	*0.03*

Multivariable model adjusted for age, sex, hemoglobin A_1c_, body mass index, systolic blood pressure and albuminuria

Italic text indicates P < 0.05

In a subgroup analysis of patients with and without known coronary heart disease, the association was both uni- and multivariably present only in patients without known coronary heart disease, Additional file 1: Tables S3 and S4.

**Fig. 1:**
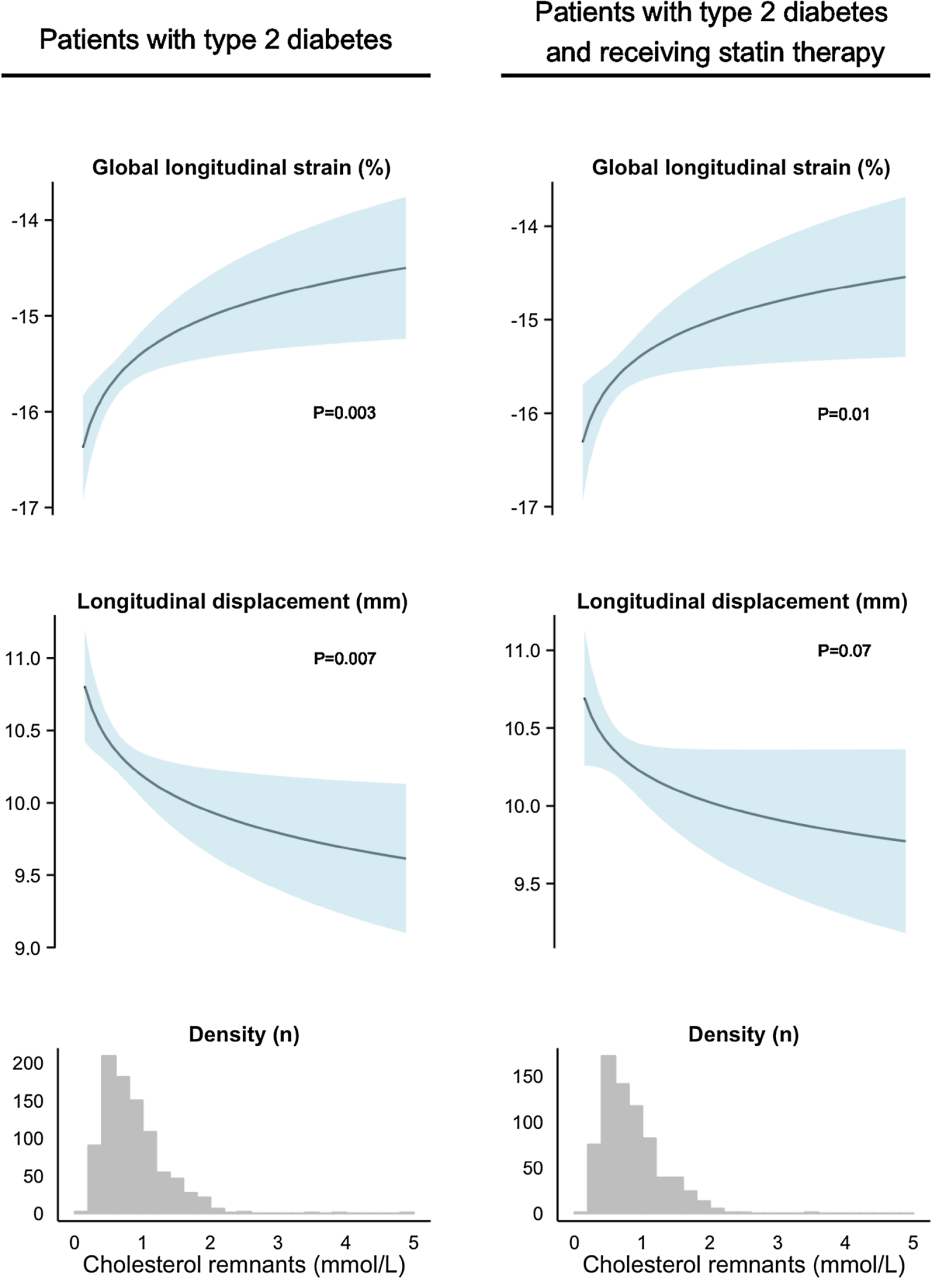
Association of cholesterol remnants levels to global longitudinal strain and longitudinal displacement in patients with type 2 diabetes and in patients with type 2 diabetes receiving statin therapy. The upper curves depict the predicted value with 95 % confidence interval of GLS and longitudinal displacement for increasing levels of cholesterol remnants on the original scale for both all patients and patients receiving statin therapy. The lower histograms are the distribution of cholesterol remnants in all patients and patients receiving statin therapy. Cholesterol remnants were calculated by total-cholesterol − HDL-cholesterol − LDL-cholesterol

**Fig. 2:**
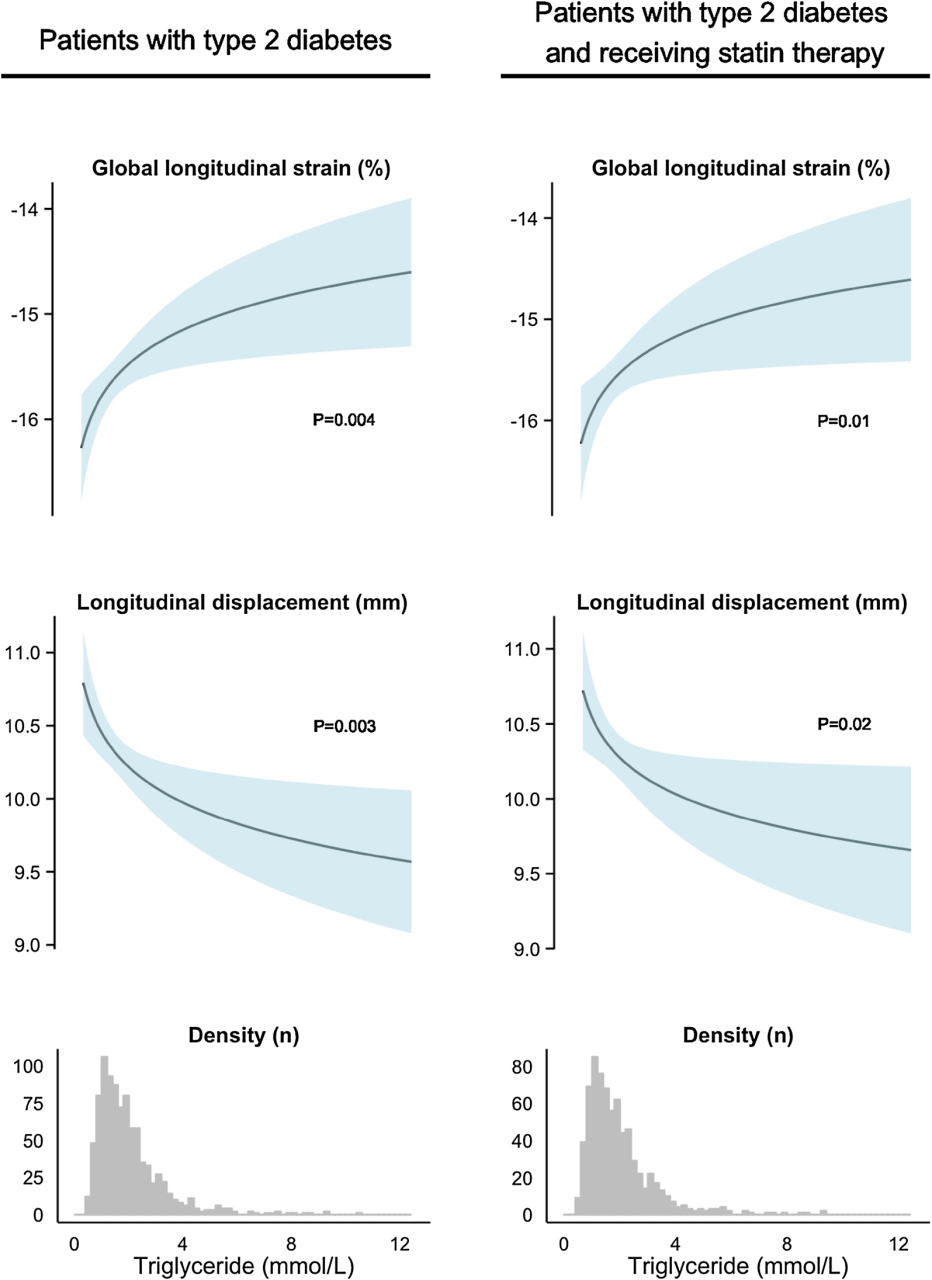
Association of triglyceride levels to global longitudinal strain and longitudinal displacement in patients with type 2 diabetes and in patients with type 2 diabetes receiving statin therapy. The upper curves depict the predicted value with 95 % confidence interval of GLS and longitudinal displacement for increasing levels of triglycerides on the original scale for both all patients and patients receiving statin therapy. The lower histograms are the distribution of triglycerides in all patients and patients receiving statin therapy

Figures 1 and 2, show the predicted value with 95 % confidence interval of GLS and longitudinal displacement for increasing levels of cholesterol remnants and triglyceride levels respectively on the original scale for both all patients and patients receiving statin therapy as well as the distribution of cholesterol remnant and triglyceride levels in all patients and patients receiving statin therapy.

## Discussion

This study is the first to demonstrate that increasing levels of cholesterol remnants and triglycerides—which are markers of cholesterol remnants—are associated with subtle changes in LV systolic function expressed as GLS and longitudinal displacement in patients with type 2 diabetes—even when receiving statin therapy. This association was not present with increasing levels of LDL-cholesterol, which was adequately treated in this patient population. So, even despite having treated LDL-cholesterol to low levels, we were able to detect a relationship between increasing levels of cholesterol remnants and triglycerides and decreasing longitudinal LV function indicating that the detrimental effect of dyslipidemia on the heart is not exhausted with intensive statin therapy. These findings support the notion of cholesterol remnants as a potential therapeutic target in addition to statin therapy in these patients.

We also found, that the association was only present in patients without known coronary heart disease, which is probably because the effect of ischemia and infarction overshadows the effect of the triglycerides and cholesterol remnants on LV myocardial function or possibly because of lack of power in this cohort.

### Dyslipidemia, LV changes and risk of heart failure

Animal models have linked cholesterol levels with changes in both diastolic and systolic performance [22, 23] and one study found an association with LV ejection fraction and HDL-cholesterol in patients with angina pectoris [24]. In a recent paper, Mochizuki et al. [25] studied the clinical features of decreased GLS in 144 patients with diabetes and found that among others hypertriglyceridemia was closely associated with low GLS. In patients with hypertension, hypercholesterolemia and low levels of HDL-cholesterol have been linked to increased LV mass [26, 27], and—in a mixed population—high levels of triglyceride were associated with increased LV mass index and decreased diastolic function expressed as lower e’, E/A ratio and longer deceleration time and isovolumetric relaxation time [28]. In accordance with these studies, we found increased LV mass with increasing levels of triglyceride (that is inversely related to HDL-cholesterol), which however, was attenuated when performing multivariable adjustment. The opposite was the case, in the diastolic measurements, where we only found changes in e’, peak E velocity and E/A ratio after multivariable adjustment. These differences may be explained by differences in patient populations and/or because of categorical cut-offs whereas we used continuous.

The influence of lipid levels on cardiac structure and incident heart failure has also been examined in the Framingham cohort. Though they did find a strong relationship between both non-HDL- and HDL-cholesterol levels and risk of heart failure in this population [29], they concluded, that there was no meaningful relationship between changes in LV structure and lipid levels. However, they did not examine any functional changes in the form of diastolic and systolic measurements [30]. In conclusion, our study is mainly in accordance with previous studies reporting a weak association of lipid levels and LV structure and diastolic function. In addition, our results suggests that the increased risk of heart failure related to dyslipidemia may be demonstrated in an early phase using sensitive measures of LV function including GLS and longitudinal displacement.

### Cholesterol remnants, triglycerides and longitudinal dysfunction as marker of early ischemic heart disease?

Remnant lipoproteins are thought to cause atherosclerosis by mechanisms similar to LDL-cholesterol, which is by entering and accumulating in the intima of the arterial wall [7, 8], ultimately leading to coronary occlusion. Consequently, a number of studies have assigned an increased risk of coronary heart disease with increasing levels of triglycerides/cholesterol remnants [13, 31, 32]. However, as this is a slowly progressing process, it is likely, that only partial occlusion of the coronary arteries is present in the early stages of the disease affecting primarily the oxygen delivery to the distal parts of the coronary arteries. Here, in the subendocardial myofiber band, is where the longitudinal myocardial fibers are situated, and hence, the longitudinal function is thought to be the first to be affected in coronary atherosclerosis [9]. Therefore, measures of longitudinal function like GLS and longitudinal displacement are considered to be sensitive markers of early signs of the impact of atherosclerosis on cardiac function [33]. This is consistent with our findings where increasing levels of both triglyceride and cholesterol remnants were associated with decreasing longitudinal function, and in this light, it is possible that subclinical atherosclerosis in patients with high levels of cholesterol remnants and triglycerides is part of the explanation to the decreased longitudinal function we found in patients with type 2 diabetes. This is further supported by Wei et al. [34], who recently proposed myocardial steatosis as a mechanistic link between diastolic dysfunction and coronary microvascular dysfunction in women.

### Cholesterol remnants, triglycerides and accumulation of myocardial triglycerides

At least one other mechanism may lead to decreased myocardial function with increasing levels of triglycerides and cholesterol remnants. In type 2 diabetes, excess availability of free fatty acids in the myocardium shifts the substrate for metabolism to almost entirely to rely on oxidation of free fatty acids, which in turn is thought to be a major cause of diabetic cardiomyopathy, that is, left ventricular impairment in patients with diabetes in the absence of coronary artery disease and hypertension [35]. Triglycerides are cleaved on the surface of lipoprotein remnants by the action of lipoprotein lipase present either in the endothelium or the intima of the coronary arteries [10]. In accordance, cardiac steatosis as measured by cardiac magnetic resonance spectroscopy has been shown to be present in patients with diabetes [36], and to be associated both with impairment in diastolic function [37] and systolic function expressed as decreased GLS with normal ejection fraction [38]. In addition, a study by Hammer et al. [39] showed that prolonged caloric restriction in obese patients with type 2 diabetes decreased myocardial triglyceride content and improved diastolic heart function. Also, this was accompanied by a decrease in fasting plasma-triglyceride and total-cholesterol levels. Taken together, these findings are very consistent with our findings that demonstrated deterioration of both diastolic (however vague) and systolic function with increasing levels of cholesterol remnants and triglyceride levels.

### Strengths and limitations

This study is strong, in that it is a large cohort of patients with type 2 diabetes which is very well characterized with clinical characteristics, laboratory values and comprehensive echocardiography. Also, sample size is sufficiently large to perform sub-analyses of patients without known heart disease and—in particular—patients receiving statin therapy. However, the study has some limitations. Though the majority of patients had non-fasting lipid measurements a minority only had fasting lipids available. However, the association persisted when excluding patients with fasting lipid measurements from the analyses. Also, as this is an observational study, it is not possible to entirely rule out residual confounding.

## Conclusions

In patients with type 2 diabetes, subtle decrease in left ventricular function is present with increasing levels of cholesterol remnants and triglyceride levels indicating an effect of these on cardiac function that is not detectable by conventional echocardiography. This association is present even in patients that are adequately treated with statin therapy. These findings support the notion of cholesterol remnants as possible therapeutic target in addition to statin therapy.

## Additional file

10.1186/s12933-016-0454-x Echocardiographic characteristics of all patients and patients receiving statin therapy.** Table S2.** Structural changes in relation to log_2_(LDL cholesterol).** Table S3.** Echocardiographic findings in patients without known coronary heart disease, n = 762.** Table S4.** Echocardiographic findings in patients with known coronary heart disease, n = 162.

## Abbreviations

BMI: body mass index
LV: left ventricular
LA: left atrial
E: peak early mitral inflow velocity
A: peak atrial mitral inflow velocity
E’: early diastolic myocardial velocity
GLS: global longitudinal strain
