# Supplementary material for: Cholesterol remnants and triglycerides are associated with decreased myocardial function in patients with type 2 diabetes
Source: Cardiovasc Diabetol. 2016 Sep 22;15:137. doi: 10.1186/s12933-016-0454-x (PMC5034540; doi:10.1186/s12933-016-0454-x)
Supplement: Supplementary file 1 — 10.1186/s12933-016-0454-x Echocardiographic characteristics of all patients and patients receiving statin therapy. Table S2. Structural changes in relation to log2(LDL cholesterol). Table S3. Echocardiographic findings in patients without known coronary heart disease, n = 762. Table S4. Echocardiographic findings in patients with known coronary heart disease, n = 162. [file 12933_2016_454_MOESM1_ESM.docx]

**Supplemental files**

Table S1. Echocardiographic characteristics of all patients and patients receiving statin therapy.

|  | All | Recieving statin therapy |
| --- | --- | --- |
|  | n=924 | n=730 |
| Left ventricular mass index (g/m2.7) | 38.8 (11.1) | 39.1 (11.0) |
| Interventricular septum diameter (mm) | 10.6 (1.8) | 10.7 (1.8) |
| Left ventricular internal diameter (mm) | 46.0 (6.2) | 46.1 (6.2) |
| Posterior wall diameter (mm) | 10.4 (1.6) | 10.4 (1.6) |
| Ejection fraction (%) | 59.6 (8.2) | 59.8 (7.8) |
| Left atrial end systolic volume (ml) | 51.3 [39.4, 64.0] | 52.0 [40.3, 64.9] |
| Left atrial end systolic volume index (ml/m2) | 24.9 [19.6, 30.6] | 25.0 [19.7, 30.8] |
| Peak E velocity (m/s) | 0.8 (0.2) | 0.8 (0.2) |
| Peak A velocity (m/s) | 0.8 (0.2) | 0.8 (0.2) |
| E deceleration time (ms) | 232 (72) | 231 (72) |
| Lateral e’ (cm/s) | 8.7 (2.5) | 8.6 (2.4) |
| Septal e’ (cm/s) | 6.7 (1.9) | 6.7 (1.8) |
| E/e’mean | 10.5 (4.2) | 10.5 (4.0) |
| E/A ratio | 0.9 (0.3) | 0.9 (0.3) |
| Longitudinal displacement (mm) | 10.3 (2.0) | 10.3 (2.0) |
| Global longitudinal strain (%) | -15.6 (2.6) | -15.6 (2.7) |

Table S2. Structural changes in relation to log_2_(LDL cholesterol)

|  | | | **Patients with type 2 diabetes** | | | |  |  | **Patients with type 2 diabetes and receiving statin therapy** | | | |  |
| --- | --- | --- | --- | --- | --- | --- | --- | --- | --- | --- | --- | --- | --- |
|  |  | Univariable | | | Multivariable | | | Univariable | | | Multivariable | | |
|  |  | β-coefficient  (std error) | | P-value | β-coefficient (std error) | P-value | | β-coefficient (std error) | | P-value | β-coefficient (std error) | P-value | |
| **Structural changes** | |  | |  |  |  | |  | |  |  |  | |
|  | Left ventricular mass index (g/m^2.7^) | -0.4 (0.7) | | 0.51 | 0.3 (0.6) | 0.65 | | -0.5 (0.8) | | 0.58 | 0.5 (0.8) | 0.53 | |
|  | Interventricular septum diameter (mm) | -0.26 (0.11) | | **0.01** | -0.12 (0.10) | 0.21 | | -0.23 (0.13) | | 0.09 | -0.10 (0.12) | 0.39 | |
|  | End diastolic internal diameter (mm) | -0.07 (0.37) | | 0.85 | 0.53 (0.37) | 0.15 | | 0.13 (0.46) | | 0.79 | 0.63 (0.45) | 0.16 | |
|  | Posterior wall diameter (mm) | -0.25 (0.09) | | **0.006** | -0.10 (0.08) | 0.22 | | -0.25 (0.11) | | **0.03** | -0.12 (0.10) | 0.25 | |
|  | Left atrial end systolic volume index (ml/m^2^) | -0.14 (0.50) | | 0.78 | 0.30 (0.51) | 0.55 | | 0.37 (0.64) | | 0.56 | 0.77 (0.63) | 0.22 | |
|  |  |  | |  |  |  | |  | |  |  |  | |
| **Diastolic changes** | | | |  |  |  | |  | |  |  |  | |
|  | Peak E velocity (m/s) | 0.002 (0.01) | | 0.89 | -0.005 (0.01) | 0.70 | | -0.02 (0.01) | | 0.26 | -0.02 (0.01) | 0.28 | |
|  | Peak A velocity (m/s) | 0.002 (0.01) | | 0.86 | -0.01 (0.01) | 0.42 | | -0.02 (0.01) | | 0.20 | -0.02 (0.01) | 0.06 | |
|  | E deceleration time (ms) | 0.00 (0.02) | | 0.86 | 0.01 (0.02) | 0.67 | | 0.00 (0.02) | | 0.82 | 0.01 (0.02) | 0.59 | |
|  | E/A ratio | -0.69 (4.40) | | 0.87 | 0.81 (4.43) | 0.85 | | -0.93 (5.48) | | 0.86 | 0.52 (5.44) | 0.92 | |
|  | Lateral e’ (cm/s) | 0.13 (0.15) | | 0.39 | 0.13 (0.14) | 0.33 | | 0.26 (0.18) | | 0.15 | 0.21 (0.17) | 0.20 | |
|  | Septal e’ (cm/s) | -0.03 (0.11) | | 0.78 | -0.07 (0.10) | 0.46 | | 0.10 (0.14) | | 0.46 | 0.03 (0.13) | 0.81 | |
|  | E/e’_mean_ | 0.20 (0.25) | | 0.44 | 0.10 (0.24) | 0.67 | | -0.32 (0.31) | | 0.30 | -0.23 (0.29) | 0.42 | |
|  |  |  | |  |  |  | |  | |  |  |  | |
| **Systolic changes** | |  | |  |  |  | |  | |  |  |  | |
|  | Ejection fraction (%) | 0.06 (0.5) | | 0.90 | -0.5 (0.5) | 0.36 | | 0.73 (0.58) | | 0.21 | 0.16 (0.59) | 0.78 | |
|  | Global longitudinal systolic strain (%) | -0.06 (0.17) | | 0.72 | -0.01 (0.18) | 0.94 | | -0.22 (0.21) | | 0.29 | -0.13 (0.22) | 0.54 | |
|  | Longitudinal displacement (mm) | 0.03 (0.12) | | 0.80 | 0.00 (0.12) | 0.98 | | 0.12 (0.15) | | 0.42 | 0.05 (0.15) | 0.71 | |
| Multivariable model adjusted for age, sex, hemoglobin A_1c_, body mass index, systolic blood pressure and albuminuria  Bold text indicates P<0.05 | | | | | | | | | | | | | |

Table S3. Echocardiographic findings in patients without known coronary heart disease, n=762

|  | | | log_2_(cholesterol remnants) | | | |  |  | log_2_(triglyceride) | | | |  |
| --- | --- | --- | --- | --- | --- | --- | --- | --- | --- | --- | --- | --- | --- |
|  |  | Univariable | | | Multivariable | | | Univariable | | | Multivariable | | |
|  |  | β-coefficient (std error) | | P-value | β-coefficient (std error) | P-value | | β-coefficient (std error) | | P-value | β-coefficient (std error) | P-value | |
| **Structural changes** | |  | |  |  |  | |  | |  |  |  | |
|  | Left ventricular mass index (g/m^2.7^) | 0.6 (0.6) | | 0.28 | -0.7 (0.5) | 0.23 | | 1.2 (0.5) | | **0.02** | -0.2 (0.5) | **0.66** | |
|  | Interventricular septum diameter (mm) | 0.23 (0.09) | | **0.01** | 0.05 (0.09) | 0.58 | | 0.30 (0.08) | | **<0.001** | 0.10 (0.08) | 0.22 | |
|  | End diastolic internal diameter (mm) | -0.46 (0.31) | | 0.14 | -0.77 (0.31) | **0.01** | | -0.28 (0.28) | | 0.33 | -0.68 (0.29) | **0.02** | |
|  | Posterior wall diameter (mm) | 0.23 (0.08) | | **0.004** | 0.06 (0.07) | 0.39 | | 0.32 (0.07) | | **<0.001** | 0.13 (0.07) | 0.07 | |
|  | Left atrial end systolic volume index (ml/m^2^) | -1.51 (0.43) | | **<0.001** | -1.46 (0.45) | **0.001** | | -1.26 (0.39) | | **0.001** | -1.29 (0.41) | **0.002** | |
|  |  |  | |  |  |  | |  | |  |  |  | |
| **Diastolic changes** | |  | |  |  |  | |  | |  |  |  | |
|  | Peak E velocity (m/s) | -0.02 (0.01) | | 0.15 | -0.03 (0.01) | **0.009** | | -0.01 (0.01) | | 0.12 | -0.03 (0.01) | **0.005** | |
|  | Peak A velocity (m/s) | -0.0003 (0.01) | | 0.98 | - 0.001 (0.01) | 0.92 | | - 0.003 (0.01) | | 0.70 | -0.002 (0.01) | 0.72 | |
|  | E/A ratio | -0.01 (0.01) | | 0.47 | -0.03 (0.01) | 0.07 | | -0.01 (0.01) | | 0.59 | -0.02 (0.01) | 0.06 | |
|  | E deceleration time (ms) | 3.14 (3.78) | | 0.41 | 4.03 (3.86) | 0.30 | | 0.23 (3.41) | | 0.95 | 1.27 (3.55) | 0.72 | |
|  | Lateral e’ (cm/s) | -0.26 (0.13) | | 0.05 | -0.29 (0.12) | **0.02** | | -0.24 (0.12) | | 0.05 | -0.27 (0.11) | **0.02** | |
|  | Septal e’ (cm/s) | -0.08 (0.10) | | 0.41 | -0.11 (0.09) | 0.22 | | -0.06 (0.09) | | 0.50 | -0.10 (0.09) | 0.26 | |
|  | E/e’_mean_ | 0.14 (0.23) | | 0.55 | -0.04 (0.22) | 0.85 | | 0.09 (0.21) | | 0.65 | -0.07 (0.20) | 0.73 | |
|  |  |  | |  |  |  | |  | |  |  |  | |
| **Systolic changes** | |  | |  |  |  | |  | |  |  |  | |
|  | Ejection fraction (%) | -0.6 (0.4) | | 0.16 | -0.3 (0.4) | 0.56 | | -0.4 (0.4) | | 0.29 | -0.1 (0.4) | 0.88 | |
|  | Global longitudinal systolic strain (%) | 0.42 (0.14) | | **0.003** | 0.31 (0.15) | **0.04** | | 0.37 (0.13) | | **0.004** | 0.27 (0.14) | **0.05** | |
|  | Longitudinal displacement (mm) | -0.26 (0.10) | | **0.01** | -0.22 (0.10) | **0.03** | | -0.23 (0.09) | | **0.01** | -0.20 (0.09) | 0.03 | |
| Multivariable model adjusted for age, sex, hemoglobin A_1c_, body mass index, systolic blood pressure and albuminuria  Bold text indicates P<0.05 | | | | | | | | | | | | | |

Table S4. Echocardiographic findings in patients with known coronary heart disease, n=162

|  | | | log_2_(cholesterol remnants) | | | |  |  | log_2_(triglyceride) | | | |  |
| --- | --- | --- | --- | --- | --- | --- | --- | --- | --- | --- | --- | --- | --- |
|  |  | Univariable | | | Multivariable | | | Univariable | | | Multivariable | | |
|  |  | β-coefficient (std error) | | P-value | β-coefficient (std error) | P-value | | β-coefficient (std error) | | P-value | β-coefficient (std error) | P-value | |
| **Structural changes** | |  | |  |  |  | |  | |  |  |  | |
|  | Left ventricular mass index (g/m^2.7^) | -0.2 (1.3) | | 0.89 | -0.01 (1.4) | 0.99 | | -0.1 (1.1) | | 0.93 | -0.2 (1.3) | 0.86 | |
|  | Interventricular septum diameter (mm) | 0.06 (0.20) | | 0.77 | -0.10 (0.21) | 0.61 | | 0.04 (0.18) | | 0.82 | -0.17 (0.19) | 0.37 | |
|  | End diastolic internal diameter (mm) | 0.42 (0.74) | | 0.57 | 0.52 (0.84) | 0.54 | | 0.50 (0.67) | | 0.45 | 0.48 (0.77) | 0.54 | |
|  | Posterior wall diameter (mm) | -0.18 (0.16) | | 0.27 | -0.17 (0.18) | 0.34 | | -0.08 (0.15) | | 0.58 | -0.12 (0.16) | 0.46 | |
|  | Left atrial end systolic volume index (ml/m^2^) | -2.42 (0.88) | | **0.007** | -1.28 (0.97) | 0.19 | | -1.95 (0.80) | | **0.02** | -1.00 (0.88) | 0.26 | |
|  |  |  | |  |  |  | |  | |  |  |  | |
| **Diastolic changes** | |  | |  |  |  | |  | |  |  |  | |
|  | Peak E velocity (m/s) | -0.01 (0.02) | | 0.64 | -0.02 (0.02) | 0.38 | | -0.01 (0.02) | | 0.58 | -0.02 (0.02) | 0.27 | |
|  | Peak A velocity (m/s) | -0.01 (0.02) | | 0.68 | 0.002 (0.02) | 0.94 | | -0.01 (0.02) | | 0.67 | -0.001 (0.02) | 0.94 | |
|  | E deceleration time (ms) | -0.01 (0.03) | | 0.82 | -0.03 (0.04) | 0.40 | | -0.01 (0.03) | | 0.68 | -0.03 (0.03) | 0.31 | |
|  | E/A ratio | 1.87 (8.52) | | 0.83 | 0.62 (9.99) | 0.95 | | 2.78 (7.54) | | 0.71 | 2.76 (8.93) | 0.76 | |
|  | Lateral e’ (cm/s) | 0.17 (0.25) | | 0.50 | -0.06 (0.25) | 0.83 | | 0.03 (0.22) | | 0.89 | -0.16 (0.23) | 0.48 | |
|  | Septal e’ (cm/s) | -0.16 (0.17) | | 0.36 | -0.37 (0.18) | **0.04** | | -0.13 (0.16) | | 0.39 | -0.33 (0.17) | 0.05 | |
|  | E/e’_mean_ | -0.05 (0.39) | | 0.91 | 0.14 (0.42) | 0.74 | | -0.01 (0.35) | | 0.97 | 0.08 (0.39) | 0.84 | |
|  |  |  | |  |  |  | |  | |  |  |  | |
| **Systolic changes** | |  | |  |  |  | |  | |  |  |  | |
|  | Ejection fraction (%) | -0.5 (1.1) | | 0.67 | -1.0 (1.2) | 0.41 | | -0.7 (1.0) | | 0.46 | -0.9 (1.1) | 0.42 | |
|  | Global longitudinal systolic strain (%) | 0.18 (0.33) | | 0.58 | 0.38 (0.38) | 0.33 | | 0.09 (0.31) | | 0.77 | 0.19 (0.36) | 0.60 | |
|  | Longitudinal displacement (mm) | -0.11 (0.24) | | 0.65 | -0.28 (0.27) | 0.30 | | -0.19 (0.21) | | 0.36 | -0.30 (0.25) | 0.23 | |
| Multivariable model adjusted for age, sex, hemoglobin A_1c_, body mass index, systolic blood pressure and albuminuria  Bold text indicates P<0.05 | | | | | | | | | | | | | |
